# Tubeimoside-I sensitizes colorectal cancer cells to chemotherapy by inducing ROS-mediated impaired autophagolysosomes accumulation

**DOI:** 10.1186/s13046-019-1355-0

**Published:** 2019-08-14

**Authors:** Jianghong Yan, Xiaoyun Dou, Jing Zhou, Yuanfeng Xiong, Ling Mo, Longhao Li, Yunlong Lei

**Affiliations:** 1 Institute of Life Science,Chongqing Medical University, Chongqing, 400016 China; 2 Department of Medical Laboratory Technology, Chongqing Medical University, Chongqing, 400016 China; 3 Department of Oncology, The First Affiliated Hospital of Chongqing Medical University, Chongqing, 400016 China; 4 Department of Biochemistry and Molecular Biology and Molecular Medicine and Cancer Research Center, Chongqing Medical University, Chongqing, 400016 People’s Republic of China

**Keywords:** Tubeimoside-I, Autophagy, ROS, AMPK, Chemosensitivity

## Abstract

**Background:**

Tubeimoside-I (TBM), a plant-derived bioactive compound, shows antitumor activity in different tumors and can enhance the efficacy of chemotherapeutic agents. However, the detail mechanism underlying remains to be elucidated.

**Methods:**

The cytotoxic potential of TBM towards CRC cells was examined by CCK8 assay, colony formation, LDH release assay, flow cytometry method and Western blots. The ROS levels, autophagy, apoptosis, chemosensitivity to 5-FU or DOX, etc. were determined between control and TBM-treated CRC cells.

**Results:**

In this study, we found that TBM could inhibit proliferation and induce apoptosis in colorectal cancer (CRC) cells. Intriguingly, TBM treatment could either promote autophagy initiation by ROS-induced AMPK activation, or block autophagy flux through inhibiting lysosomal hydrolytic enzymes, which leaded to massive impaired autophagylysosomes accumulation. Administration of autophagy initiation inhibitor (3-MA or selective ablation of autophagy related proteins) relieves TBM-induced CRC suppression, while combination use of autophagy flux inhibitor chloroquine (CQ) slightly augments TBM-induced cell death, suggesting that impaired autophagylysosomes accumulation contributes to TBM-induced growth inhibition in CRC cells. Notably, as an autophagy flux inhibitor, TBM works synergistically with 5-fluorouracil (5-FU) or doxorubicin (DOX) in CRC suppression.

**Conclusion:**

Together, our study provides new insights regarding the anti-tumor activity of TBM against CRC, and established potential applications of TBM for CRC combination therapies in clinic.

**Electronic supplementary material:**

The online version of this article (10.1186/s13046-019-1355-0) contains supplementary material, which is available to authorized users.

## Background

Although the dissemination of colonoscopy with polypectomy has led to a decline in the incidence and mortality rates, colorectal cancer (CRC) still is the third most diagnosed cancers and the second leading causes of cancer death among both men and women worldwide, with over 1.8 million new colorectal cancer cases and 881,000 deaths in 2018 [1, 2]. If CRC patients can be diagnosed and treated at localized stage, 5-year survival rate of which is about 90%, that will decline to 71 and 14% for patients diagnosed with regional and distant-stage disease [1, 3]. Adjuvant chemotherapy is the main treatment strategy for advanced CRC, however, a subset of patients eventually become relapsed because of acquired drug resistance to the current commonly used chemotherapeutic drugs and the effectiveness of chemotherapy has often been limited [4, 5]. Hence at present, development of new drugs for colorectal cancer is an urgent need.

The stem tuber of *Bolbostemma paniculatum (Maxim.) Franquet (Cucurbitaceae)* is a traditional Chinese medicine named as *Rhizoma Bolbostemmatis* (Chinese name“Tu Bei Mu”) [6, 7]. It was listed in the Supplement to the Compendium of Materia Medica in Qing Dynasty for treating breast cancer, acute mastitis, inflammation and snake venoms [6, 7]. Tubeimoside-I (TBM), a triterpenoid saponin, is isolated from *Rhizoma Bolbostemmatis* and shows antitumor activity in different tumor such as promyelocytic leukemia, lung cancer, cervical cancer, nasopharyngeal carcinoma, esophageal carcinoma with low toxicity [6, 7]. TBM could trigger a mitochondrial-related apoptotic pathway and cell cycle arrest in cervical carcinoma, ovarian cancer, choriocarcinoma and glioma [8–11]. TBM can also inhibit the growth and invasion of CRC cells [12]. More interestingly, tubeimosides are effective in combination therapies, particularly at targeting drug-resistant cancerous cells [13]. However, the underlying mechanisms of its anti-tumor activity have not been fully clarified so far, especially in CRC.

Autophagy is a highly conserved catabolic process during which de novo-formed membrane-enclosed vesicles engulf damaged or senescent organelles and transport to lysosomes for degradation and recycling in response to nutrient starvation or metabolic stress [14, 15]. Autophagy plays an important role in the regulation of cancer progression and in determining the response of tumor cells to stress induced by chemotherapy [14, 15]. Four functional forms of autophagy induced by chemotherapy have been described to date: the cytoprotective autophagy which facilitates the resistance of cancer cells to chemotherapeutic drugs, cytotoxic autophagy which promotes cell death, cytostatic autophagy which prolongs growth inhibition as well as reduced clonogenic survival, and nonprotective autophagy which don’t affect drug or radiation sensitivity [16]. Considering the crucial roles during chemotherapy, targeting the autophagy process has recently attracted considerable attention to develop novel antitumor therapeutics via pharmacological modulation of autophagy. Tubeimoside-I can induce cytoprotective autophagy in human breast cancer cells in vitro, while promote cytotoxic autophagy in cervical cancer cells [17, 18]. However, the role of autophagy in TBM-treated CRC cells remains largely unexplored, let alone the underlying mechanisms.

In this study, we found that TBM inhibited the growth of CRC cells by stimulating apoptosis. Interestingly, TBM elicits autophagy by ROS-induced activation of AMPK and blocks autophagic flux by impairing the degradation of the autophagolysosome, which contributes to TBM-induced anti-cancer activity. Notably, as an autophagy modulator, TBM synergistically suppresses CRC cell growth with 5-FU or DOX. These findings provide the evidences for the use of TBM as a new therapeutic agent against CRC, especially in combination chemotherapy.

## Material and methods

### Cell culture

The SW480 and SW620 cell lines were purchased from the American Type Culture Collection (ATCC), HCT116 and RKO cell lines were purchased from Shanghai cell bank. All cell lines were cultured according to the guidelines and were maintained in DMEM (Gibco) supplemented with 100 U/mL penicillin (Sigma), 10 mg/L streptomycin (Sigma), and 10% serum (Hyclone) in a humidified incubator at 37 °C under 5% CO_2_ atmosphere.

### Reagents and antibodies

The following primary antibodies were used: Lamp1 (Santa Cruz Biotechnology), LC3 (MBL International Corporation), p62 (MBL International Corporation), PARP 1(Cell Signaling Technology), PRKAA/AMPK (Cell Signaling Technology), phosphor-PRKAA/ AMPK(Cell Signaling Technology), Beclin1 (Cell Signaling Technology), ATG5 (Cell Signaling Technology), CASP9 (Cell Signaling Technology), CASP3 (Cell Signaling Technology) and Cleaved-CASP3 (Cell Signaling Technology). TBM (BP1415) was purchased from Phytopurify Biotechnology. 3-methyladenine (3-MA), chloroquine (CQ) and N-acetyl-L-cysteine (NAC) were purchased from Sigma-Aldrich. Doxorubicin (DOX, HY-15142A) and 5-fluorouracil (5-FU, HY-90006) were from MedChem Express.

### Transfection

All siRNAs were designed using BLOCK-iT™ RNAi Designer (Invitrogen) and synthesized by GenePharma (Shanghai, China). The sequences of the siRNAs used are listed in Additional file 1: Table S1. Cells were transfected with siRNAs using Lipofectamine RNAiMax (Invitrogen) according to the manufacturer’s instruction.

Dominant-negative AMPK (DN-PRKAA1/DN-AMPKα1) plasmid was obtained as described previously [19]. The plasmids were transfected into the indicated cells using Lipofectamine 2000 (Invitrogen) according to the manufacturer’s instruction.

### Immunofluorescence

Cells grown on coverslips that treated with TBM for 24 h were fixed with 4% paraformaldehyde (Sigma) for 30 min and then washed three times with PBS. Fixed cells were permeabilized with 0.4% Triton 100 and 5% BSA for 1 h at 37 °C. For staining, cells were incubated with primary antibodies for 12 h at 4 °C, followed by incubation with secondary antibodies (Thermos Fisher Scientific) for 1 h at 37 °C. Finally, Nuclei were stained with DAPI for 10 min. For autophagy flux studies, cells were transfected with GFP-RFP-LC3 for 24 h and then treated with TBM for another 24 h. Images were captured using a confocal microscopy (Carl Zess Micromaging).

### Cell viability and proliferation assays

As described previously [20], CCK8 assay was performed to determine cell viability. Briefly, cells were seeded in 96-well plates at a density of 5000 cells. After treatment, CCK8 (Dojindo, Kamimashiki-gun, Kumamoto, Japan; CK04) were added and incubated for 40 min. The absorbance value was then determined at 450 nm.

The long-term effects of TBM on CRC cell proliferation were analyzed with a colony formation assay. After treatment, 1500–2000 cells were seeded in six-well plates and the medium was changed every 3 days. After two weeks, cells were fixed with 4% paraformaldehyde (Sigma) for 30 min and stained with Crystal Violet for another 30 min, then the colonies were washed three times and taken photos.

### Immunoblot and immunoprecipitation

Cells were lysed with RIPA buffer (50 mM Tris base, 1.0 mM EDTA, 150 mM NaCl, 0.1% SDS, 1% Triton X-100, 1% sodium deoxycholate, 1 mM PMSF) supplemented with protease inhibitor cocktail (Sigma) and then protein lysates were centrifuged and boiled with loading buffer. For immunoprecipitation, Whole cell lysates were prepared in RIPA buffer (40 mM Tris-HCl, pH 7.5, 150 mM NaCl, 0.5% Nonidet P-40, cocktail, 5% glycerol, 10 mM NaF) and incubated with 1 μg of antibody overnight at 4 °C. Next day, Sepharose protein A/protein G beads were added for 2 h. The immune-complexes were then centrifuged and washed 3 times using RIPA buffer. All lysates were quantified by the BCA Protein Assay (Thermo Fisher Scientific) and analysed by SDS–PAGE.

### Transmission electron microscopy

Transmission electron microscopy assay was used to visualize autophagic vesicles. After 24-h TBM treatment, SW480 and HCT116 cells were fixed in glutaraldehyde (Sigma) and ultrathin sections were prepared using a sorvall MT5000 microtome. Then, the sections were stained by lead citrate and /or 1% uranyl acetate and visualized by Philips EM420 electron microscopy.

### Flow cytometry

As described previously [21], the indicated cells treated with TBM for 24 h were harvested and washed three times with PBS, then resuspended and incubated with PI-ANXA5 solution (KeyGEN Biotech). Apoptosis was analyzed with a FACSCalibur flow cytometer (Becton Dickinson, San Jose, CA USA).

### Reactive oxygen species (ROS) measurement

Intracellular ROS level was detected by staining cells with 2′, 7′-dichlorofluorescein diacetate (DCFH-DA) (GENMED, GMS10016.2) according to the manufacturer’s instructions. The DCFH-DA signal was measured with a FACSCalibur flow cytometer or a Molecular Devices SPECTRAMAX M5 fluorimeter (490 nm excitation and 530 nm emission).

### Data analysis and statistics

Data were expressed as means ± s.d. All experiments were performed at least three times. Statistical analysis was performed with GraphPad Prism 6.0 software. Statistical differences between groups were determined using two-tailed Student’s t-test. Significance was designated as follows: *, *P* < 0.05, **, *P* < 0.01, ***, *P* < 0.001.

## Results

### TBM inhibits proliferation and induces apoptosis in CRC cell

**Fig. 1:**
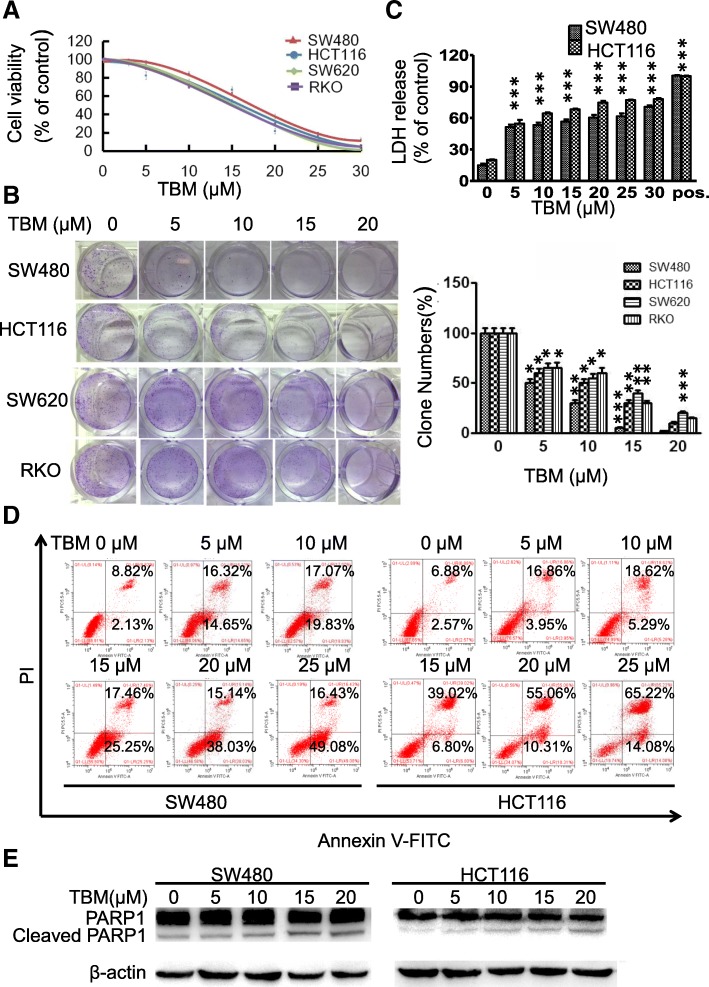
TBM inhibits proliferation and induces apoptosis in CRC cells. **a**. SW480, HCT116, SW620 and RKO cells were treated with the indicated concentration of TBM for 24 h and cell viability was determined by CCK8 kit. **b**. Cell proliferation rate was analyzed by clone formation assay. SW480, HCT116, SW620 and RKO cells were treated with the indicated concentration of TBM for 24 h, after treatment, cells were seeded into 6-well plates for two weeks and colony numbers were quantified. **c.** Analysis of dehydrogenase (LDH) release in SW480 and HCT116 cells subjected to indicated concentration of TBM. Cell lysates serve as positive control. **d**. SW480 and HCT116 cells treated with 10 μM TBM for 24 h were fixed, stained with Annexin V/PI, and then analyzed by flow cytometry. **e**. Immunoblot analysis of PARP1 in HCT116 and SW480 cells. Data are means ± s.d. and are representative of 3 independent experiments. *, *P* < 0.05; **, *P* < 0.01; ***, *p* < 0.001

To validate whether TBM exhibited antitumor effect against CRC, CCK8 assay was performed to assess the cell viability in response to TBM treatment in different human CRC cell lines. As shown in Fig. 1a, TBM treatment for 24 h markedly decreased the cell viability of various CRC cell lines (SW480, HCT116, SW620 and RKO) in a dose-dependent manner. Consistently, the proliferation of CRC cells was significantly inhibited under TBM treatment, as evidenced by reduced colony formation (Fig. 1b). To further verify its anti-CRC activity, lactate dehydrogenase (LDH) release assay was performed and the results showed that TBM treatment exhibited marked cytotoxicity in SW480 and HCT116 cells (Fig. 1c). The initiation of apoptosis is largely involved in the antitumor activity of chemotherapeutic drugs, thus TUNEL assay and Annexin-V/PI double staining flow cytometry method were used to evaluate whether apoptosis is involved in TBM-induced cytotoxicity in CRC. As shown as Fig. 1d and Additional file 1 :Figure S1A, TBM could induce a prominent apoptotic effect in CRC cells with a dose-dependent manner. The pro-apoptotic effect of TBM was further supported by increased levels of cleaved PARP1, CASP9 and CASP3, the specific and sensitive markers of apoptosis, in TBM-treated cells (Fig. 1e and Additional file 1 :Figure S1B). Taken together, these results indicated that TBM exhibited a significant antitumor effect in CRC cells.

### TBM activates autophagy in CRC cell

**Fig. 2:**
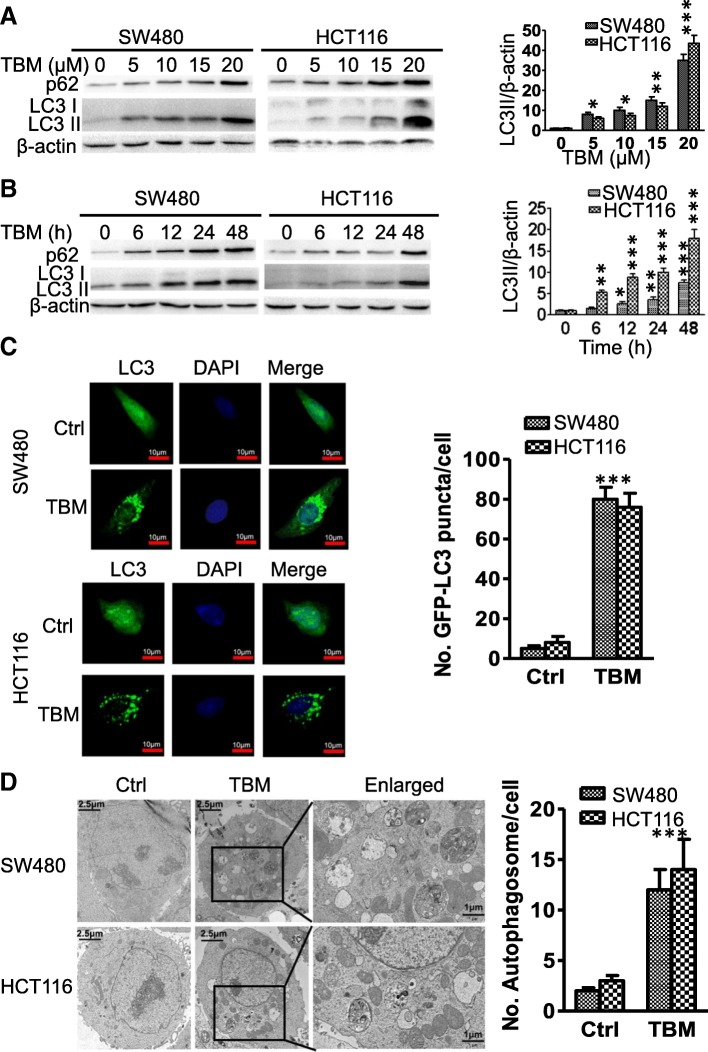
TBM induces autophagy in CRC cells. **a**. Immunoblot analysis of LC3 and p62 expression in SW480 and HCT116 cells treated with indicated concentrations of TBM for 24 h. **b**. Time course analysis of LC3 and p62 expression in SW480 and HCT116 cells treated with TBM (10 μM) for 6, 12, 24, 48 h by immunoblotting. **c**. SW480 and HCT116 cells were transfected with GFP-LC3 for 48 h, and then treated with 10 μM TBM for another 24 h. GFP-LC3 puncta was shown and quantitated by immunofluorescence analysis. Scale bar, 10 μm. **d**. Autophagic vesicles were detected by transmission electron microscope in SW480 and HCT116 cells treated as in (C). Scale bar, 2.5 or 1 μm. Data are means ± s.d. and are representative of 3 independent experiments. *, *P* < 0.05; **, *P* < 0.01; ***, *p* < 0.001

Accumulated evidence has shown that autophagy was usually activated by chemotherapeutic drugs and contributed to antitumor effect [14, 15]. To test whether autophagy is regulated by TBM in CRC cells, we first examined the processing of LC3-I to its PE-conjugated LC3-II, which is one of the most widely accepted methods for monitoring autophagy [22]. As indicated in Fig. 2a and b, TBM treatment promoted the turnover of LC3-I to lipidated LC3-II in a dose- and time-dependent manner in CRC cells. In line with this, accumulation of GFP-LC3 puncta was dramatically increased in CRC cells treated with TBM by fluorescence images (Fig. 2c). Furthermore, transmission electron microscopy experiment also showed that the appearance of double-membraned autophagosomes was frequently observed in CRC cancer cells treated with TBM (Fig. 2d).

**Fig. 3:**
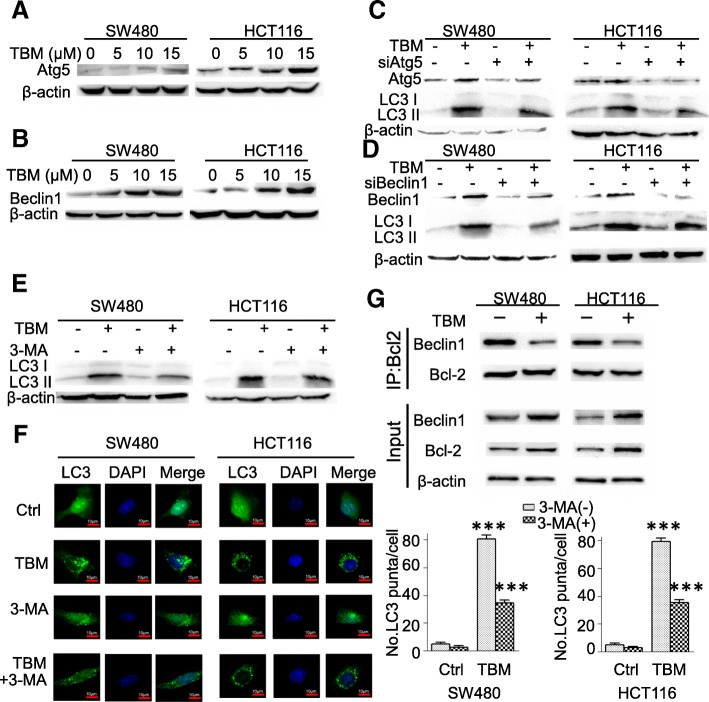
TBM induces autophagy initiation in CRC cells. **a**. Immunoblot analysis of Atg5 expression in SW480 and HCT116 cells treated with indicated concentrations of TBM for 24 h. **b**. Immunoblot analysis of Beclin 1 expression in HCT116 and SW480 cells treated with indicated concentrations of TBM for 24 h. **c**. Immunoblot analysis of LC3 and Atg5 expression in CRC cells transfected with siNC or siAtg5 for 24 h, followed by treatment with or without 10 μM TBM for another 24 h. **d**. Immunoblot analysis of LC3 and Beclin 1 expression in HCT116 and SW480 cells transfected with siNC or siBeclin 1 for 24 h, followed by treatment with or without 10 μM TBM for another 24 h. **e**. SW480 and HCT116 cells were treated with vehicle, TBM (10 μM), 3-MA (5 mM), or in combination for 24 h. Immunoblot analysis was used to detect the expression of LC3. **f**. SW480 and HCT116 cells were transfected with GFP-LC3 for 48 h, and then were treated as in (E). The LC3 puncta by immunofluorescence analysis was shown and quantitated. Scale bar, 10 μm. **g**. Co-immunoprecipitation analysis of the interaction between Beclin1 and Bcl-2 in SW480 and HCT116 cells treated with or without 10 μM TBM for 24 h. ***, *p* < 0.001

Next, we examined the expression levels of Atg5 and Beclin 1, two autophagy-related proteins, which is correlated with the extent of autophagosome formation. Results showed that TBM induced the expression of Atg5 and Beclin 1 in a dose-dependent manner in CRC cells (Fig. 3a-b). While silencing the expression of Beclin 1 or Atg5 using siRNA partially blocked LC3 lipidation in TBM-treated cells (Fig. 3c-d). Coadministration of 3-MA, a class III PI3K inhibitor, with TBM failed to induce autophagy (Fig. 3e-f). In addition, it has been reported that the diminished interaction of Beclin 1 with Bcl-2 are key events in the initiation process of autophagy [22]. As shown in coimmunoprecipitation assay, TBM treatment decreased the binding of Beclin 1 with Bcl-2 (Fig. 3g). Collectively, our data suggest that TBM induce autophagy initiation and autophagosome accumulation in CRC cells.

### ROS/AMPK axis contributes to TBM-induced autophagy initiation

**Fig. 4:**
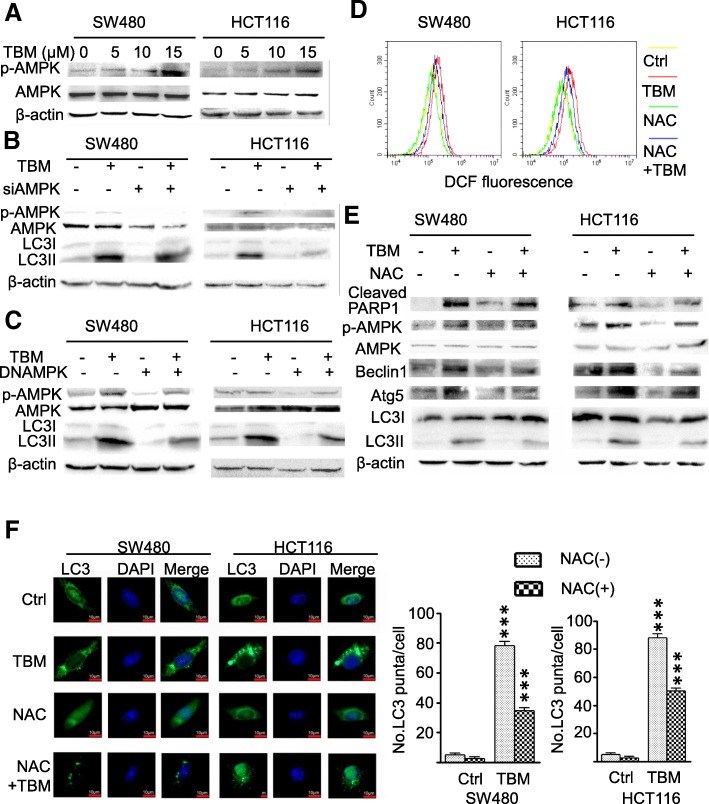
TBM-induced autophagy is dependent on ROS/AMPK activation in CRC cells. **a.** SW480 and HCT116 cells were treated with indicated concentrations of TBM. The total and phosphorylation levels of AMPK were examined by immunoblotting. **b**. Immunoblot analysis of LC3, AMPK and p-AMPK expression in SW480 and HCT116 cells transfected with siNC or siAMPK for 24 h, followed by treatment with or without 10 μM TBM for another 24 h. **c**. SW480 and HCT116 cells were transfected with Vector or DN-AMPK for 24 h, followed by treatment with or without 10 μM TBM for another 24 h. Immunoblot analysis of LC3, AMPK and p-AMPK expression in CRC cells. **d**-**e**. SW480 and HCT116 cells were treated with vehicle, TBM (10 μM), NAC (5 mM), or in combination for 24 h. ROS was detected by flow cytometric analysis (D), and the levels of cleaved-PARP1, p-AMPK, AMPK, Beclin1, Atg5 and LC3 were examined by immunoblotting (E). **F.** SW480 and HCT116 cells were transfected with GFP-LC3 for 48 h, and then treated as (D-E). GFP-LC3 puncta was shown and quantitated by immunofluorescence analysis. Scale bar, 10 μm. ***, *p* < 0.001

Previous studies have shown that TBM promote cell death mainly by triggering a mitochondrial-related apoptotic pathway [13], which may decrease the production of energy and then activate AMPK signaling pathway to initiate autophagy [23]. To ascertain this hypothesis, we detected the phosphorylation status of AMPK to validate whether AMPK was involved in TBM-induced autophagy. As shown in Fig. 4a, TBM treatment resulted in activation of AMPK as evidenced by increased phosphorylation levels of AMPK in a dose-dependent manner in TBM-treated cells. To determine whether the activation of AMPK was involved in TBM-induced autophagy, we transfected a siRNA target AMPK to inhibit AMPK expression or domain negative AMPK (DNAMPK) to inhibit AMPK activation. As shown in Fig. 4b-c, inhibition of AMPK prominently inhibited elevation of LC3 lipidation in TBM-treated cells. Dysfunction of mitochondria-induced activation was mainly involved in ROS generation, thus, we determined whether TBM could increase cellular ROS levels. As shown as Fig. 4d, treatment of TBM dramatically increased cellular ROS levels in SW480 and HCT116 cells. Accumulating evidence has shown ROS in response to cancer treatment can promote autophagy by activating various signaling pathways including AMPK signaling. Next, we would like to identify the role of ROS in TBM-induced autophagy initiation. We found that combinatorial treatment of TBM with ROS scavenger N-Acetyl-cysteine (NAC) resulted in AMPK inhibition, reduced LC3-II turnover and accumulation of GFP-LC3 punta, decreased expression of Beclin1 and Atg5 (Fig. 4e and f). Collectively, these results suggest that activation of ROS/AMPK signaling pathway is required for TBM-induced autophagy in CRC cells.

### TBM impairs autophagy flux in CRC cell by inhibiting proteolytic activity of lysosome

**Fig. 5:**
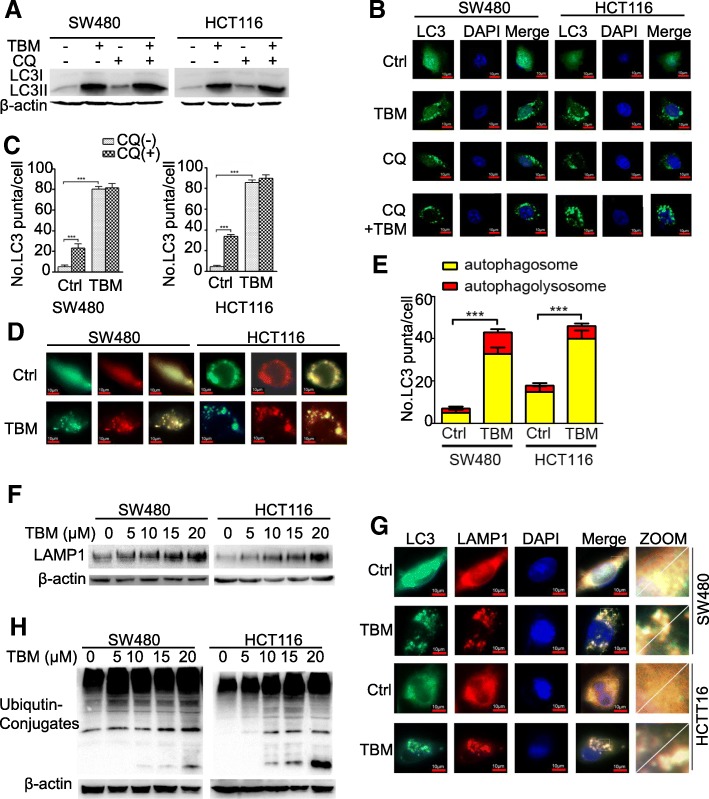
TBM inhibits autophagy flux in CRC cells. **a**. SW480 and HCT116 cells were treated with vehicle, TBM (10 μM), CQ (5 μM), or in combination for 24 h. Immunoblot analysis was used to detect the expression of LC3. **b**. The accumulation of LC3 puncta was examined by fluorescent analysis of cells transfected with GFP-LC3 plasmids and treated with CQ (5 μM) in the presence or absence of TBM (10 μM) for 24 h. Scale bar, 10 μm. **c**. The number of LC3 puncta was quantitated. **d**. Fluorescence analysis of cells transiently transfected with tandem mRFP-GFP-tagged LC3 and treated with vehicle or TBM (10 μM). Scale bar, 10 μm. **e**. The ratio of red puncta indicating autophagolysosome (GFP^−^/RFP^+^) versus yellow puncta indicating autophagosome (GFP^+^/RFP^+^) was quantitated. **f**. SW480 and HCT116 cells were treated with indicated concentrations of TBM. The expression of LAMP1 was examined by immunoblotting. **g**. Immunofluorescence analysis of the co-localization of endogenous LC3 and LAMP1 in SW480 and HCT116 cells treated with vehicle or TBM (10 μM) for 24 h. Scale bar, 10 μm. **h**. Immunoblot of ubiquitin in SW480 and HCT116 cells treated with TBM in indicated concentrations for 24 h. ***, *p* < 0.001

To determine whether TBM can also induce complete autophagy flux in CRC cells, we firstly examined the protein levels of p62, a well-known autophagic substrate. However, the results showed that endogenous p62 protein expression were notably increased dose- and time-dependently after TBM stimulation (Fig. 2a-b), suggesting that autophagic flux may be inhibited by TBM. To further confirm this result, we measured autophagy flux by monitoring the conversion of LC3-I to LC3-II in the presence of chloroquine (CQ), a lysosomal inhibitor. The result showed that combinatorial treatment of TBM with CQ did not further significantly enhanced LC3-II turnover in CRC cells (Fig. 5a-c). Using a tandem monomeric RFP-GFP–tagged LC3, we also found that TBM treatment induced notable formation of yellow fluorescent autophagosomes and slight increase of red fluorescent autophagolysosomes (Fig. 5d-e). These results demonstrated that TBM impairs autophagy flux in CRC cells.

Autophagy flux signified autophagosomes fuse with lysosomes for further degradation, thus dysfunction of lysosomes may play an important role in inhibition of TBM-induced autophagy flux. We firstly detected the expression of lysosome- associated membrane protein (LAMP) 1, a marker for mature lysosome. As shown in Fig. 5f, TBM significantly up-regulated of LAMP1 in a dose dependent manner, indicating that TBM promote lysosomal mature. We subsequently examined whether autophagosome-lysosome fusion was affected by TBM by assessing the colocalization of endogenous LC3 with LAMP1. However, our results also showed that TBM treatment induced obvious colocalization of LC3 with LAMP1, suggesting the fusion of autophagosome with lysosome (Fig. 5g). Finally, we compared the lysosomal hydrolytic enzymes after TBM treatment by examining the level of ubiquitinated proteins. Interestingly, ubiquitinated protein conjugates was increased following TBM treatment (Fig. 5h). These results suggested that TBM impairs autophagy flux by inhibiting proteolytic activity of lysosome.

### ROS-induced impaired autophagolysosomes accumulation contributes to TBM-induced cell death

**Fig. 6:**
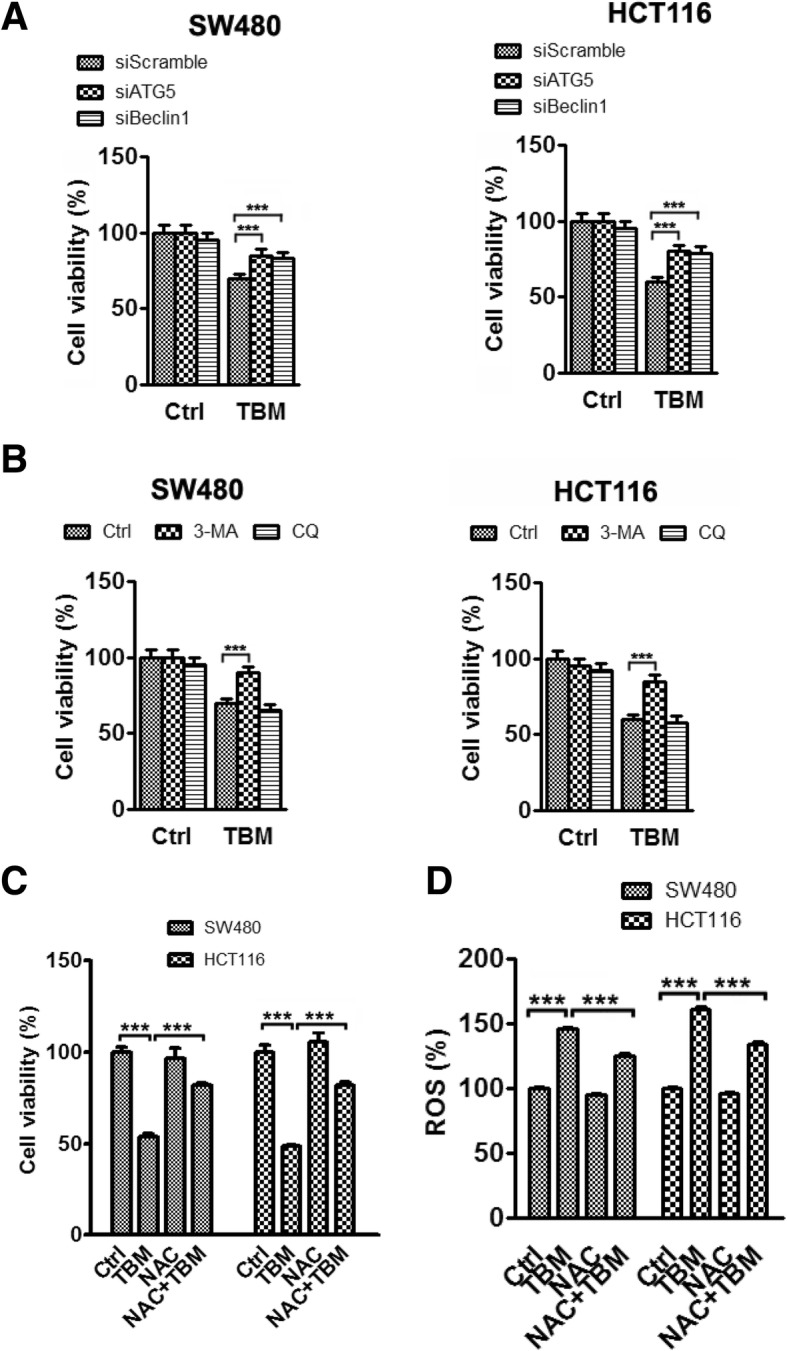
ROS-induced impaired autophagolysosomes accumulation contributes to TBM-induced cell death. **a**. Cells were transfected with siScramble, siATG5 or siBeclin1 for 24 h, and then treated with 10 μM TBM for another 24 h. Cell viability was detected by CCK8 assay. **b**. Cells were treated with 10 μM TBM in the absence or presence of 3-MA (5 mM) or CQ (5 μM). Cell viability was determined by CCK8 assay**. c.** Cells were treated with 10 μM TBM in the absence or presence of NAC (5 mM). Cell viability was determined by CCK8 assay. **d**. Cells were treated as in (C) and ROS level was analyzed by DCFH-DA staining via a fluorescence microplate Reader (Thermos scientific). ***, *p* < 0.001

Impaired autophagolysosomes accumulation is harmful to cancer cells and may lead to cell death. To evaluate whether autophagy was involved in the anti-CRC effect of TBM, we transfected CRC cells with Beclin1 or ATG5 siRNA, followed by TBM treatment. Cell viability was measured with CCK8 assay and the pro-apoptotic effect was examined by Annexin V/PI staining measured with flow cytometry. These data demonstrated that reduction in ATG5 or Beclin1 levels rescued the cell viability and inhibited the cell apoptosis caused by TBM (Fig. 6a and Additional file 1 :Figure S2A). Furthermore, similar results were obtained by using the autophagic inhibitors, 3-MA (Fig. 6b and Additional file 1 :FigureS2B). However, combination use of TBM with CQ slightly exacerbated TBM-induced cell death (Fig. 6b and Additional file 1 :FigureS2B). Next, we also elucidated the role of ROS accumulation in TBM-induced growth suppression. As shown in Fig. 6c-d, combinatorial treatment of TBM with ROS scavenger (NAC) inhibited TBM-induced growth suppression. In addition, treatment of NAC also reduced the pro-apoptotic effect of TBM, as evidenced by flow cytometry and levels of cleaved PARP1 (Fig. 4e and Additional file 1 :FigureS2C). These data indicated that TBM-induced autophagolysosome accumulation by ROS contributes to cell death in CRC cells.

### TBM enhances the chemotherapeutic response of CRC cells response to 5-FU and DOX

**Fig. 7:**
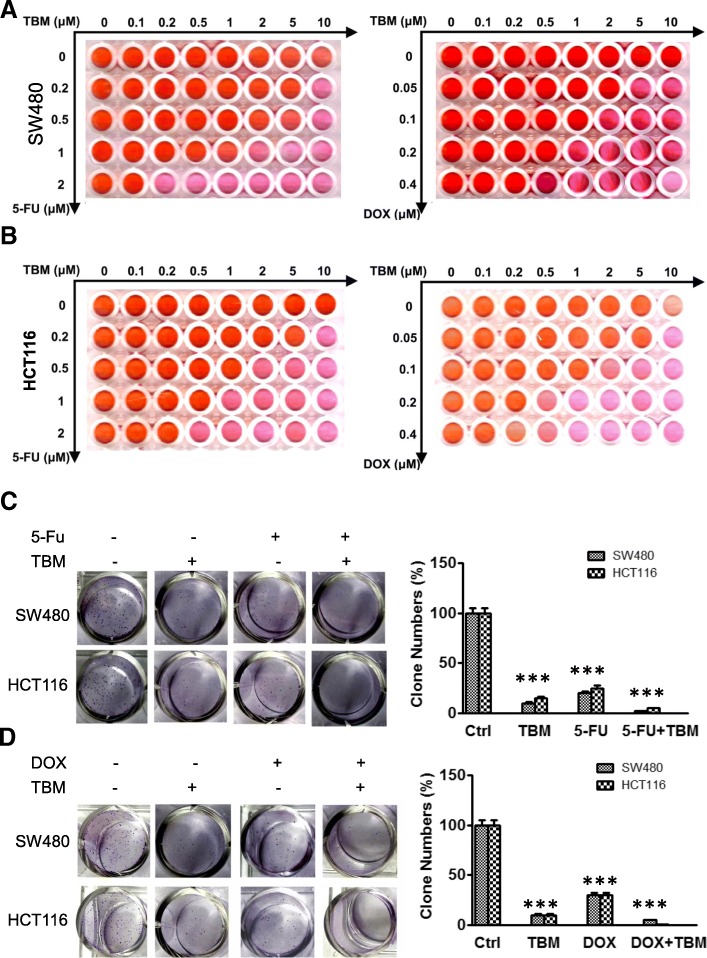
TBM enhances the chemotherapeutic response of CRC cells response to 5-FU and DOX. **a**-**b**. SW480 (A) and HCT116 (B) cells were treated with indicated concentrations of 5-fluorouracil (5-FU) or doxorubicin (DOX) in the absence or presence of indicated concentrations TBM for 24 h and CCK8 assays were performed to assess cell viability. **c**-**d**. Cell proliferation rate was analyzed by clone formation assay. SW480 and HCT116 cells were treated with 5-fluorouracil (5-FU, 2 μM) (C) or doxorubicin (DOX, 0.4 μM) (D) in the absence or presence of indicated concentrations TBM (10 μM) for 24 h, after treatment, cells were seeded into 6-well plates for two weeks and colony numbers were quantified. ***, *P* < 0.001

Previously studies have found that pharmacological modulation of autophagy can merit exploration as a therapeutic strategy for cancer treatment and can increase sensitivity of cancer cells to other chemotherapeutic agents which usually induces protective autophagy [14–16]. As a specific autophagy flux inhibitor, we wonder if TBM can also enhance the chemosensitivity of CRC cells to 5-FU or DOX. Firstly, we determine the antitumor effect of 5-FU and DOX to CRC cell lines with a variety of concentration. As shown as Fig. 7a-b, the cell growth of CRC cells can be inhibited by 5-FU and DOX, however the effect is not obvious as the concentration increased. To obtain the best result of TBM in combination 5-FU and DOX, CRC cells were combinational treated of TBM with 5-FU or DOX in different concentration. The result reveals that 2 μM 5-FU combined with 0.2 μM or 0.5 μM TBM exhibits cost-effective anticancer effect in SW480 cells and HCT116 cells, respectively, while 0.2 μM DOX combined with 1 μM TBM markedly decreased the cell viability in both SW480 cells and HCT116 cells. Consistently, significantly lower cell growth and clone formation are observed in the combination arm with this treatment (Fig. 7c-d). Together, these results indicated that TBM effectively sensitizes CRC cells to 5-FU and DOX treatment.

## Discussion

Recently, TBM has been found to have considerable potential to inhibit various types of cancer [13]. Multiple mechanisms of TBM have been uncovered recently, including the modulation of multiple enzyme activities and signaling pathways [13]. It is of great interest to reveal novel activities and mechanisms of TBM in cancer treatment. In the present study, we demonstrated that TBM exhibited potent antitumor effect on CRC. TBM inhibited the growth of CRC via inhibition of cell proliferation and induction of apoptosis. Interestingly, we also found that TBM was a potent autophagy modulator in CRC cells. TBM initiates autophagy by activating ROS-AMPK signaling and impairs autophagy flux through inhibiting lysosomal proteolysis activity, leading to the rough accumulation of autophagolysosomes in CRC cells. Meanwhile, our findings indicated that accumulation of immature autophagolysosomes by TBM triggered apoptotic cell death. Our results also provided the support that low concentration of TBM synergizing with 5-FU and DOX shows preferential cytotoxicity compared with treatment alone.

Autophagy is a tightly-regulated catabolic process for clearing damaged or long-lived proteins and organelles, and plays important roles in response to various stresses including cancer treatment [14–16]. The role of autophagy in response to chemotherapy is multifaceted [14–16]. On one hand, autophagy usually play a supportive role to hamper the anti-cancer effectiveness and induce drug resistance by preserving the organelle function and eliminating cellular waste products [14–16]. On the other hand, autophagy also can be pro-death or inhibit proliferation to benefit the anticancer outcome [14–16]. In this study, we found that TBM could significantly induce autophagy initiation and promote the fusion of autophagosome with lysosome, while impairs the degradation ability of autophagolysosome, leading to the robust accumulation of autophagolysosomes and autophagic cargo proteins (p62). The inhibition of autophagic flux is harmful to cancer cells in chemotherapy and has potential for clinical benefit. Indeed, inhibition of autophagy initiation could counteract TBM-induced cytotoxicity, while impeding autophagy flux by CQ would further promote cell death slightly in CRC cells, suggesting autophagy account for the anti-cancer activity of TBM in CRC cells.

Accumulating evidence has shown that autophagy can give cancer cells resistant ability to cancer treatment including chemotherapy and radiotherapy, whereas inhibition of autophagy could enhance the efficacy of anticancer agents [14, 24, 25]. Targeting the autophagy process has been regarded as a novel therapeutic approach, and autophagy inhibitors including chloroquine (CQ) and its derivative hydroxychloroquine (HCQ) have been applied in clinic [14, 24, 25]. Thus, development of novel autophagy modulator is worthwhile to be exploited. The classical chemotherapeutic drug 5-FU and DOX have exhibited compromised treatment outcome in CRC treatment due to protective autophagy initiation [26–28]. Thus, several efforts have been made to explore new combinational therapies with autophagy inhibitor [26–28]. TBM has been reported to enhance the efficacy of many chemotherapeutic agents in cancer cells. For example, TBM has the capability to increase the sensitivity of cisplatin (CDDP)-resistant human ovarian cancer A2780/DDP cells and Hela cells toward CDDP-induced cell death [18, 29], while the combination of TBM with fuzi extracts (Radix Aconiti Lateralis Preparata) exerted synergistic effects on breast cancer MDA-MB-231 and SKBR3 cells proliferation and migration [30]. Therefore, we determined the possibility that TBM addition might sensitive CRC cells to 5-FU or DOX treatment. The present study shows that low-dose TBM in combination 5-FU or DOX exhibit good synthetic lethal effect in CRC cells, supporting that combinational treatment of TBM with conventional anticancer therapies may warrant consideration for clinical trials by modulation of autophagy.

Reactive oxygen species (ROS) is a collective term that encompasses one or more unpaired electrons of oxygen, including superoxide (O_2_^−^), hydrogen peroxide (H_2_O_2_) and the hydroxyl radical (HO•) [31, 32]. Excessive production of ROS cause biomolecules damage and even cell demise, whereas moderate levels of ROS can act as a second-messenger to regulate pro-survival pathways [31, 32]. ROS have been widely reported to play a pivotal role in apoptosis and autophagy upon cancer treatment [33]. Major sources of cellular ROS are generated from mitochondrial respiration, NADPH oxidases (NOXs), peroxisomal β-oxidation and endoplasmic reticulum (ER) [31, 32]. TBM has been shown to induce mitochondrial damage and ROS generation in several cancer cells including hepatoma HepG2 cells, prostate cancer DU145 cells, lung cancer A549 and PC9 cells, as well as glioma U251 cells, which was involved in TBM-induced cell death [10, 13, 34–36]. In this study, we also found that TBM can promote ROS production, which in turn induces autophagy initiation by activating AMPK.

AMPK has been well characterized to play an essential role in the induction of autophagy response to cancer treatment and could be regulated by redox modification under oxidative stress [19, 37, 38]. AMPK can trigger autophagy through regulation of early autophagic events, such as activating the proautophagic VPS34 complex by phosphorylating S91/S94 in beclin1, releasing ULK1 from mTORC1 or directly activating ULK1 by phosphorylation [19, 37, 38]. AMPK is a metabolic checkpoint and regulated by energy supply, and mitochondria are the most important organelles for energy production [19, 37, 38]. TBM induced apoptosis mainly via mitochondrial pathway which will damage the mitochondria and decrease the production of energy. These may lead to AMPK activation and subsequently autophagy initiation to protect cells [13]. Our study reveals that TBM strongly increases Thr172 phosphorylation of AMPK in a dose-dependent manner, which is crucial for the initiation of autophagy in TBM-treated CRC cells.

## Conclusions

In summary, we demonstrate that TBM exerts strong cytotoxicity by inducing apoptosis in this study. Moreover, we revealed that TBM could promote impaired autophagolysosomes accumulation by both enhancing autophagy initiation and disrupting the autophagy flux, which is contributed to the anticancer activity of TBM. In addition, as a specific autophagy modulator, TBM could act as one attractive drug in combining with other chemotherapy agents to treat CRC cells for overcoming drug resistance inducing by protective autophagy. Our findings provide a bold therapeutic strategy to potential use of TBM as an adjuvant for further cancer treatment, especially in CRC.

## Additional file

Additional file 1: **Figure S1.** TBM promotes apoptosis of CRC cells. **Figure S2.** ROS-induced impaired autophagolysosomes accumulation contributes to TBM-induced apoptosis. **Table S1.** List of Small Interference RNA Sequences (PDF 1173 kb)

## Data Availability

All data and materials can be provided upon request (email: leiyunlong@126.com).

## Abbreviations

3-MA: 3-methyladenine
5-FU: 5-fluorouracil
AMPK: Adenosine monophosphate-activated protein kinase
ATG: Autophagy-related
Bcl-2: B-cell lymphoma-2
CDDP: Cisplatin
CQ: chloroquine
CRC: Colorectal cancer
DOX: Doxorubicin
GFP: Green fluorescent protein
HCQ: Hydroxychloroquine
LAMP: Lysosomal-associated membrane protein
LC3: Microtubule-associated protein 1 light chain 3
LDH: Lactic dehydrogenase
NAC: N-acetyl-L-cysteine
p62: Sequestosome 1
PARP1: Poly (ADP-ribose) polymerase 1
RFP: Red fluorescent protein
ROS: Reactive oxygen species
siRNA: Small interfering RNA
TBM: Tubeimoside I
