# Supplementary material for: Tubeimoside-I sensitizes colorectal cancer cells to chemotherapy by inducing ROS-mediated impaired autophagolysosomes accumulation
Source: J Exp Clin Cancer Res. 2019 Aug 14;38:353. doi: 10.1186/s13046-019-1355-0 (PMC6694658; doi:10.1186/s13046-019-1355-0)
Supplement: Supplementary file 1 — Figure S1. TBM promotes apoptosis of CRC cells. Figure S2. ROS-induced impaired autophagolysosomes accumulation contributes to TBM-induced apoptosis. Table S1. List of Small Interference RNA Sequences (PDF 1173 kb) [file 13046_2019_1355_MOESM1_ESM.pdf]

Supplementary figure legends

Figure S1. TBM promotes apoptosis of CRC cells

A. SW480 and HCT116 cells were treated with indicated concentrations of TBM for 24 h, and apoptosis index was determined by TUNEL assay. Scale bars: 100 µm. **B.** Immunoblot analysis of PARP1, CASP3 and CASP9 in HCT116 and SW480 cells. ***, $P < 0.001$.

Figure S2. ROS-induced impaired autophagolysosomes accumulation contributes to TBM-induced apoptosis

A. Cells were transfected with siScramble, siATG5 or siBeclin1 for 24 h, and then treated with 10 µM TBM for another 24 h. Cell apoptosis was detected by Annexin V/PI staining measured with flow cytometry. **B.** Cells were treated with 10 µM TBM in the absence or presence of 3-MA (5 mM) or CQ (5 µM). Cell apoptosis was determined by Annexin V/PI staining measured with flow cytometry. **C.** Cells were treated with 10 µM TBM in the absence or presence of NAC (5 mM). Cell apoptosis was determined by Annexin V/PI staining measured with flow cytometry.

Figure S1

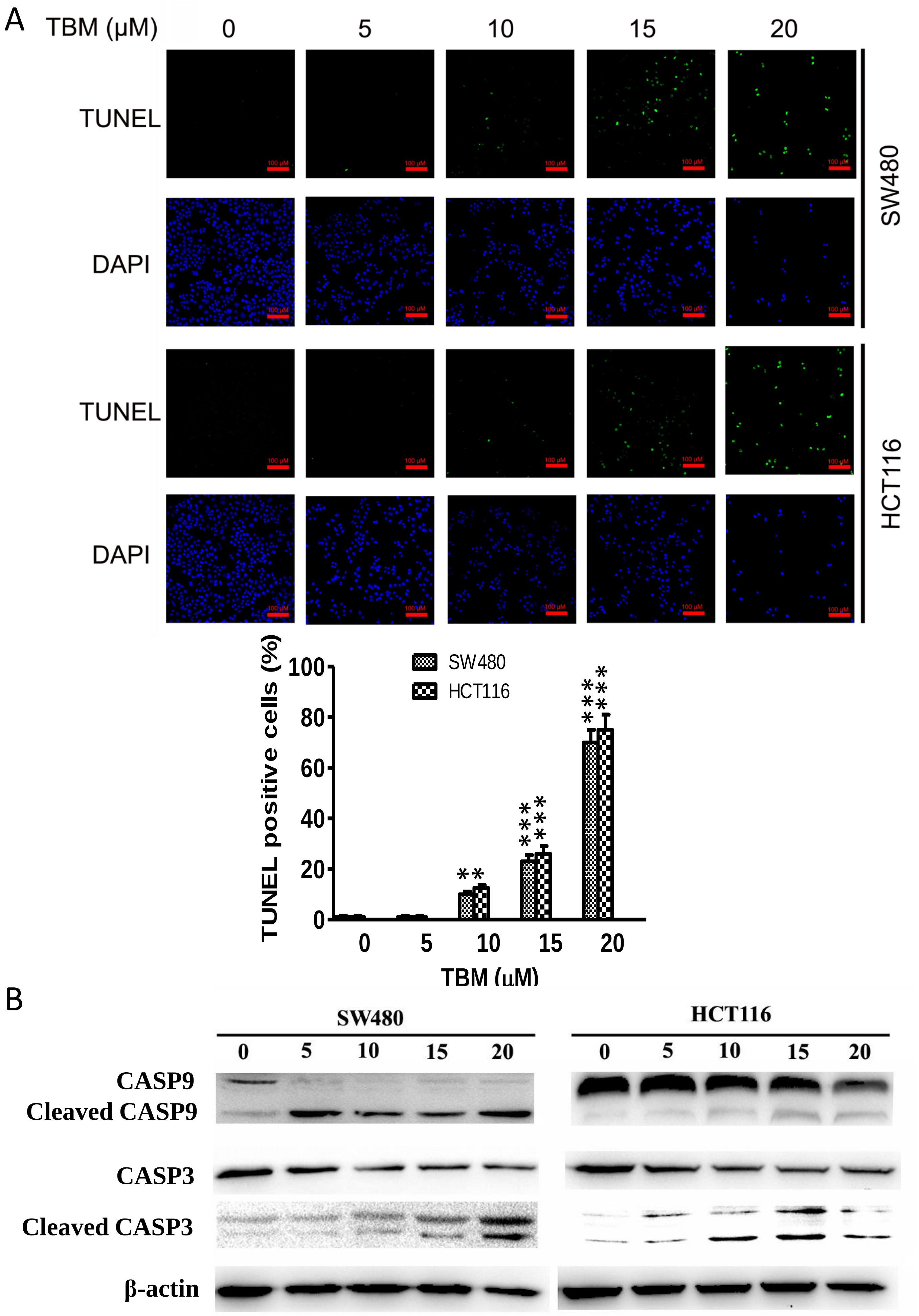
A
TBM (μM)
0
5
10
15
20
TUNEL
DAPI
TUNEL
DAPI
SW480
HCT116
SW480
HCT116
TUNEL positive cells (%)
100
80
60
40
20
0
**
***
***
***
***
0
5
10
15
20
TBM (μM)
B
SW480
HCT116
0
5
10
15
20
0
5
10
15
20
CASP9
Cleaved CASP9
CASP3
Cleaved CASP3
β-actin

Figure.S2

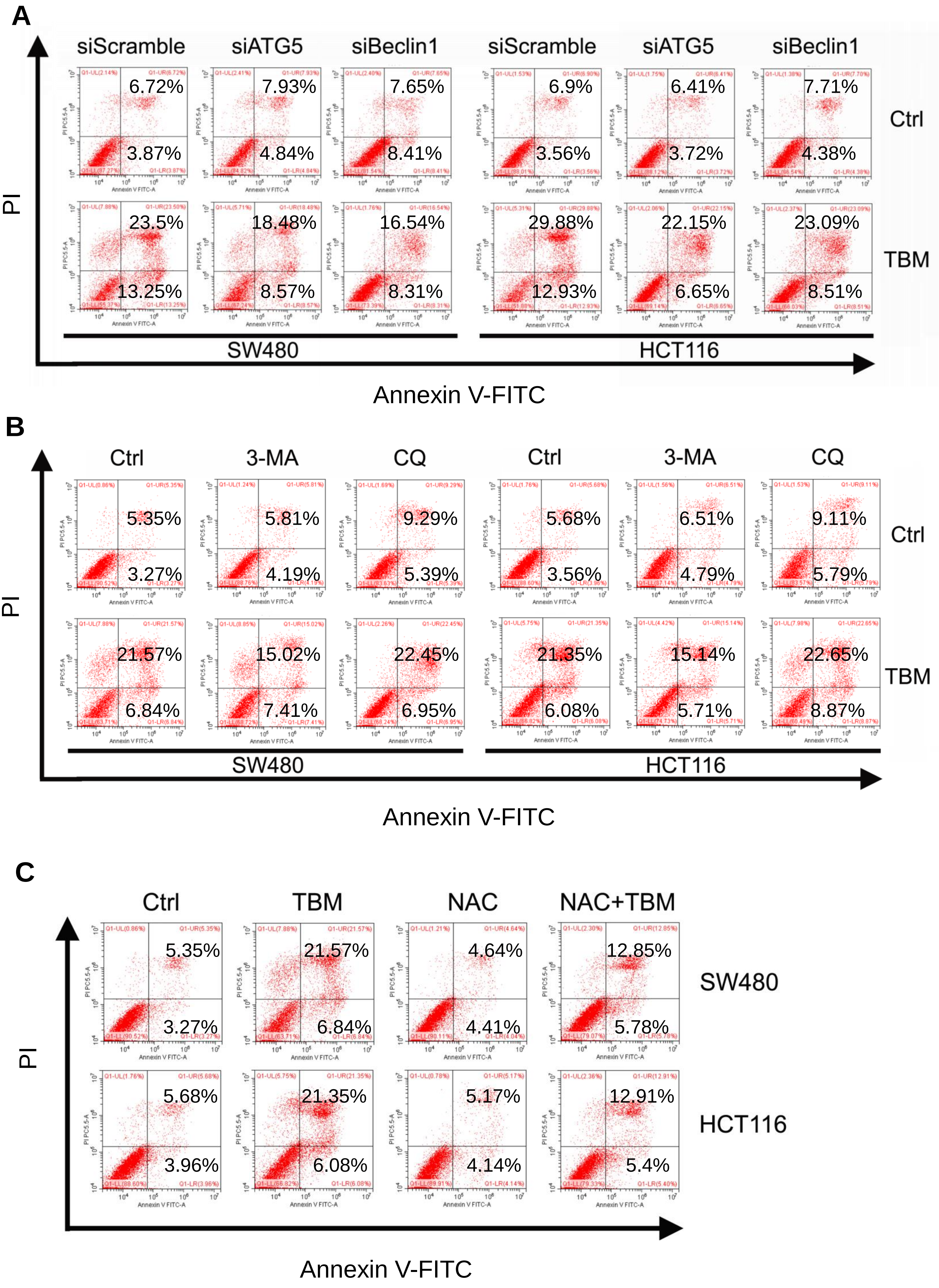
A
siScramble
siATG5
siBeclin1
siScramble
siATG5
siBeclin1
6.72%
3.87%
7.93%
4.84%
7.65%
8.41%
6.9%
3.56%
6.41%
3.72%
7.71%
4.38%
Ctrl
23.5%
13.25%
18.48%
8.57%
16.54%
8.31%
29.88%
12.93%
22.15%
6.65%
23.09%
8.51%
TBM
SW480
HCT116
PI
Annexin V-FITC
B
Ctrl
3-MA
CQ
Ctrl
3-MA
CQ
5.35%
3.27%
5.81%
4.19%
9.29%
5.39%
5.68%
3.56%
6.51%
4.79%
9.11%
5.79%
Ctrl
21.57%
6.84%
15.02%
7.41%
22.45%
6.95%
21.35%
6.08%
15.14%
5.71%
22.65%
8.87%
TBM
SW480
HCT116
PI
Annexin V-FITC
C
Ctrl
TBM
NAC
NAC+TBM
5.35%
3.27%
21.57%
6.84%
4.64%
4.41%
12.85%
5.78%
SW480
5.68%
3.96%
21.35%
6.08%
5.17%
4.14%
12.91%
5.4%
HCT116
PI
Annexin V-FITC

Supplementary Table 1. List of Small Interference RNA Sequences

Gene name	siRNA sense sequences
BECLIN 1	5'-AAGAUAGUGGCAGAAAAUCTT-'3
ATG5	5'-GACGUUGGUAACUGACAAATT-'3
NC	5'-UUCUCCGAACGUGUCACGUUU-'3
